# (3-Ethyl-6,7-dimeth­oxy­naphthalen-1-yl)(phen­yl)methanone

**DOI:** 10.1107/S1600536812004734

**Published:** 2012-02-10

**Authors:** Karuppusamy Sakthivel, Kannupal Srinivasan, Sampath Natarajan

**Affiliations:** a School of Chemistry, Bharathidasan University, Thiruchirapalli, Tamil Nadu 620 024, India; b Department of Advanced Technology Fusion, Konkuk University, 1 Hwayang-dong, Gwangjin-gu, Seoul 143 701, Republic of Korea

## Abstract

The asymetric unit of the title mol­ecule, C_21_H_20_O_3_, contains two crystallographically independent mol­ecules, *A* and *B*, which differ in the orientation of the ethyl group substituted on the naphthalene system; the dihedral angles between the ethyl group and the naphthalene system are 7.4 (3) and 68.1 (3)°, respectively, for mol­ecules *A* and *B*. The dihedral angles between the benzoyl and naphthalene groups are 64.7 (7) and 69.4 (8)°, respectively, for mol­ecules *A* and *B*. The crystal structure features four aromatic π–π stacking interactions [centroid–centroid distances = 4.181 (1), 3.891 (1), 4.423 (1) and 4.249 (1) Å].

## Related literature

For the biological activities of naphthalene compounds, see: Dekoning *et al.* (2003 ▶); Alvarez *et al.* (2007 ▶). For related crystal structures, see: Watanabe *et al.* (2010 ▶); Thenmozhi *et al.* (2008 ▶). For ring conformational analysis, see: Cremer & Pople (1975 ▶); Nardelli (1995 ▶).
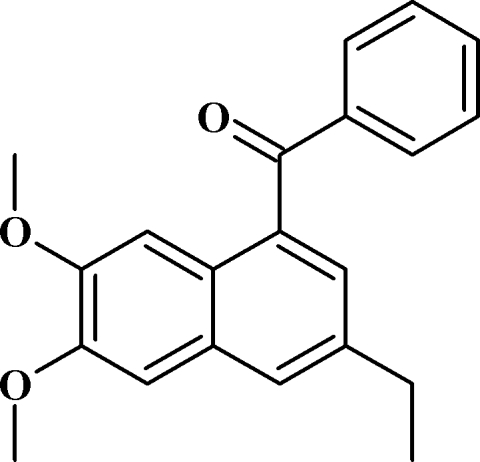


## Experimental

### 

#### Crystal data

C_21_H_20_O_3_
*M* *_r_* = 320.37
Triclinic, 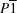

*a* = 9.9012 (2) Å
*b* = 11.3431 (2) Å
*c* = 16.0701 (3) Å
α = 100.170 (1)°
β = 90.487 (1)°
γ = 98.373 (1)°
*V* = 1756.46 (6) Å^3^
*Z* = 4
Mo *K*α radiation
μ = 0.08 mm^−1^
*T* = 293 K
0.12 × 0.08 × 0.06 mm

#### Data collection

Bruker SMART APEX CCD area-detector diffractometer
Absorption correction: part of the refinement model (Δ*F*) (*XABS2*; Parkin *et al.*, 1995 ▶) *T*_min_ = 0.869, *T*_max_ = 1.483
33428 measured reflections
7706 independent reflections
4854 reflections with *I* > 2σ(*I*)
*R*_int_ = 0.034

#### Refinement

*R*[*F* ^2^ > 2σ(*F* ^2^)] = 0.062
*wR*(*F* ^2^) = 0.214
*S* = 1.05
7706 reflections
433 parameters
H-atom parameters constrained
Δρ_max_ = 0.63 e Å^−3^
Δρ_min_ = −0.32 e Å^−3^

### 

Data collection: *SMART* (Bruker, 2004 ▶); cell refinement: *SMART*; data reduction: *SMART*; program(s) used to solve structure: *SHELXS97* (Sheldrick, 2008 ▶); program(s) used to refine structure: *SHELXL97* (Sheldrick, 2008 ▶); molecular graphics: *ORTEP-3* (Farrugia, 1997 ▶); software used to prepare material for publication: *PLATON* (Spek, 2009 ▶).

## Supplementary Material

Crystal structure: contains datablock(s) I, global. DOI: 10.1107/S1600536812004734/aa2044sup1.cif

Structure factors: contains datablock(s) I. DOI: 10.1107/S1600536812004734/aa2044Isup2.hkl

Supplementary material file. DOI: 10.1107/S1600536812004734/aa2044Isup3.cml

Additional supplementary materials:  crystallographic information; 3D view; checkCIF report

## Figures and Tables

**Fig. 1.**
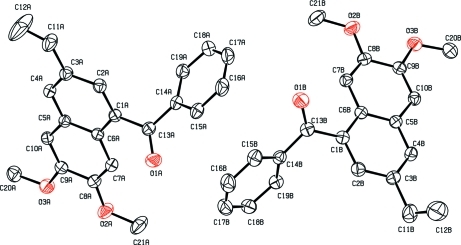
ORTEP diagram of the title molecule with the atom numbering scheme. Displacement ellipsoids are drawn at 30% probability level. H-atoms were removed for clarity.

**Fig. 2.**
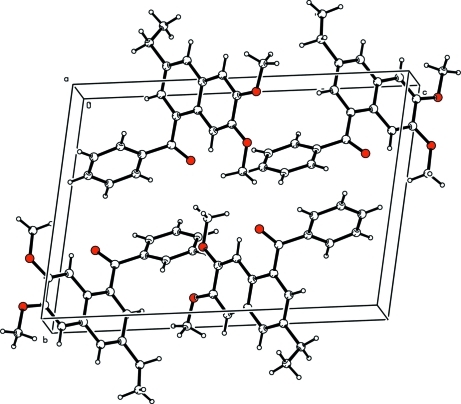
Packing diagram of the title compound viewed down the a axis.

## supplementary crystallographic information

### Comment

Polysubstituted napthalenes exhibit a wide range of biological activities such as antiviral, anti-diabetic, anti-malarial and anti-tumor activities (Dekoning *et al.*, 2003). Naphthylphenstatin (dimethoxy benzoylnaphthalene), a compound similar to the title compound, is one of the naphthalene compounds which has the activity of tubulin polymerization inhibition and cytotoxic activities (Alvarez *et al.*, 2007).

The asymmetric unit of the title compound contains two crystallographically independent molecules (Fig. 1). The corresponding bond lengths and angles of the two molecules agree with each other, and are comparable to those observed in the structures of 2,7-dimethoxy-1-(4-nitrobenzoyl)-naphthalene (Watanabe *et al.*, 2010) and naphthalene-2,3-diylbis(2-thienyl)-methanone (Thenmozhi *et al.*, 2008). The two independent molecules differ in the orientations of ethyl units with respect to the naphthalene ring system. The dihedral angle of these ethyl units and naphthalene rings A and B respectively are 7.4 (3) and 68.1 (3)°. The dihedral angles between the benzoyl group and naphthalene ring system are 64.7 (7) and 69.4 (8)° for molecules A and B, respectively.

The title molecule do not show any classical hydrogen bonds (Fig. 2). But the molecules are stabilized in the unit cell packing with help of weak π–π interactions {[*Cg*1···*Cg*2] = 4.181 (1) Å (1 - *x*, 2 - *y*, -*z*); [*Cg*2···*Cg*2] = 3.891 (1) Å (1 - *x*, 2 - *y*, -*z*); [*Cg*3···*Cg*4] = 4.423 (1) Å (-*x*, -*y*, 1 - *z*) and [*Cg*4···*Cg*4] = 4.249 (1) Å (-*x*, -*y*, 1 - *z*); here, *Cg* is the centroid of the benzene rings, *Cg*1 = C1A–C6A, *Cg*2 = C5A–C10A, *Cg*3 = C1B–C6B and *Cg*4 = C5B–C10B}.

### Experimental

The title compound was synthesized in 83% yield (0.1 g) by heating a mixture of 4,5-dimethoxy-2-phenylethynylbenzaldehyde (0.1 g, 0.3 mmol) and n-butyraldehyde (0.027 g, 0.37 mmol) in 1,2-dichloroethane for 3 h under reflux. The resulting product was recrystallized from methanol which produced light brown color crystals.

### Refinement

The terminal 12 A atom shows higher thermal motion due to free rotation which reflects in abnormal bond length (C11A—C12A). H atoms were positioned geometrically and refined using a riding model with C—H = 0.93 Å for aromatic H, C—H = 0.97 Å for methylene H and 0.96 Å for methyl H atoms. The *U*_iso_ parameters for H atoms were constrained to be 1.5Ueq of the carrier atom for the methyl H atoms and 1.2Ueq of the carrier atom for the remaining H atoms.

### Crystal data

**Table:**

C_21_H_20_O_3_	*Z* = 4
*M**_r_* = 320.37	*F*(000) = 680
Triclinic, *P*1	*D*_x_ = 1.211 Mg m^−^^3^
*a* = 9.9012 (2) Å	Mo *K*α radiation, λ = 0.71073 Å
*b* = 11.3431 (2) Å	Cell parameters from 7706 reflections
*c* = 16.0701 (3) Å	θ = 1.8–27.1°
α = 100.170 (1)°	µ = 0.08 mm^−^^1^
β = 90.487 (1)°	*T* = 293 K
γ = 98.373 (1)°	Needle, pale-brown
*V* = 1756.46 (6) Å^3^	0.12 × 0.08 × 0.06 mm

### Data collection

**Table:**

Bruker SMART APEX CCD area-detector diffractometer	7706 independent reflections
Radiation source: fine-focus sealed tube	4854 reflections with *I* > 2σ(*I*)
Graphite monochromator	*R*_int_ = 0.034
ω scans	θ_max_ = 27.1°, θ_min_ = 1.8°
Absorption correction: part of the refinement model (Δ*F*) (*XABS2*; Parkin *et al.*, 1995)	*h* = −12→12
*T*_min_ = 0.869, *T*_max_ = 1.483	*k* = −14→14
33428 measured reflections	*l* = 0→20

### Refinement

**Table:**

Refinement on *F*^2^	Primary atom site location: structure-invariant direct methods
Least-squares matrix: full	Secondary atom site location: difference Fourier map
*R*[*F*^2^ > 2σ(*F*^2^)] = 0.062	Hydrogen site location: inferred from neighbouring sites
*wR*(*F*^2^) = 0.214	H-atom parameters constrained
*S* = 1.05	*w* = 1/[σ^2^(*F*_o_^2^) + (0.1046*P*)^2^ + 0.3934*P*] where *P* = (*F*_o_^2^ + 2*F*_c_^2^)/3
7706 reflections	(Δ/σ)_max_ < 0.001
433 parameters	Δρ_max_ = 0.63 e Å^−^^3^
0 restraints	Δρ_min_ = −0.32 e Å^−^^3^

### Special details

**Table:**

Geometry. All s.u.'s (except the s.u. in the dihedral angle between two l.s. planes) are estimated using the full covariance matrix. The cell s.u.'s are taken into account individually in the estimation of s.u.'s in distances, angles and torsion angles; correlations between s.u.'s in cell parameters are only used when they are defined by crystal symmetry. An approximate (isotropic) treatment of cell s.u.'s is used for estimating s.u.'s involving l.s. planes.
Refinement. Refinement of *F*^2^ against ALL reflections. The weighted *R*-factor *wR* and goodness of fit *S* are based on *F*^2^, conventional *R*-factors *R* are based on *F*, with *F* set to zero for negative *F*^2^. The threshold expression of *F*^2^ > σ(*F*^2^) is used only for calculating *R*-factors(gt) *etc*. and is not relevant to the choice of reflections for refinement. *R*-factors based on *F*^2^ are statistically about twice as large as those based on *F*, and *R*-factors based on ALL data will be even larger.

### Fractional atomic coordinates and isotropic or equivalent isotropic displacement parameters (Å^2^)

**Table:**

	*x*	*y*	*z*	*U*_iso_*/*U*_eq_	
O1A	0.2799 (2)	0.67052 (17)	0.10690 (11)	0.0917 (6)	
O2A	0.6797 (2)	0.71872 (16)	−0.07109 (12)	0.0927 (6)	
O3A	0.80443 (17)	0.93243 (15)	−0.06671 (10)	0.0763 (5)	
C1A	0.4076 (2)	0.85790 (19)	0.17247 (13)	0.0578 (5)	
C2A	0.3801 (2)	0.9548 (2)	0.23037 (14)	0.0636 (5)	
H2A	0.3179	0.9410	0.2719	0.076*	
C3A	0.4420 (2)	1.0739 (2)	0.22952 (14)	0.0656 (5)	
C4A	0.5398 (2)	1.09145 (18)	0.17097 (13)	0.0598 (5)	
H4A	0.5845	1.1694	0.1709	0.072*	
C5A	0.5745 (2)	0.99481 (17)	0.11092 (12)	0.0532 (4)	
C6A	0.5062 (2)	0.87582 (18)	0.10996 (12)	0.0543 (5)	
C7A	0.5410 (2)	0.78111 (19)	0.04753 (14)	0.0630 (5)	
H7A	0.4963	0.7025	0.0452	0.076*	
C8A	0.6388 (2)	0.8033 (2)	−0.00900 (14)	0.0655 (5)	
C9A	0.7086 (2)	0.9229 (2)	−0.00690 (13)	0.0600 (5)	
C10A	0.6768 (2)	1.01583 (18)	0.05192 (12)	0.0562 (5)	
H10A	0.7227	1.0938	0.0534	0.067*	
C11A	0.3969 (4)	1.1763 (2)	0.2907 (2)	0.1063 (11)	
H11A	0.3015	1.1764	0.2765	0.128*	
H11B	0.3996	1.1538	0.3461	0.128*	
C12A	0.4551 (8)	1.2875 (4)	0.3001 (4)	0.272 (5)	
H12A	0.4093	1.3368	0.3422	0.408*	
H12B	0.4503	1.3156	0.2473	0.408*	
H12C	0.5491	1.2929	0.3177	0.408*	
C13A	0.3238 (2)	0.7369 (2)	0.17281 (14)	0.0641 (5)	
C14A	0.2884 (2)	0.69894 (18)	0.25523 (14)	0.0615 (5)	
C15A	0.1637 (2)	0.6265 (2)	0.26026 (17)	0.0752 (6)	
H15A	0.1058	0.6011	0.2126	0.090*	
C16A	0.1261 (3)	0.5923 (2)	0.3359 (2)	0.0930 (9)	
H16A	0.0414	0.5463	0.3397	0.112*	
C17A	0.2131 (4)	0.6259 (3)	0.4058 (2)	0.0963 (9)	
H17A	0.1866	0.6036	0.4569	0.116*	
C18A	0.3399 (3)	0.6928 (2)	0.40032 (17)	0.0895 (8)	
H18A	0.4003	0.7123	0.4470	0.107*	
C19A	0.3771 (3)	0.7307 (2)	0.32593 (15)	0.0728 (6)	
H19A	0.4615	0.7776	0.3229	0.087*	
C20A	0.8778 (3)	1.0491 (2)	−0.06818 (17)	0.0832 (7)	
H20A	0.9422	1.0438	−0.1125	0.125*	
H20B	0.9257	1.0799	−0.0148	0.125*	
H20C	0.8152	1.1027	−0.0782	0.125*	
C21A	0.6212 (4)	0.5960 (3)	−0.0752 (2)	0.1215 (13)	
H21A	0.6593	0.5463	−0.1210	0.182*	
H21B	0.5241	0.5878	−0.0844	0.182*	
H21C	0.6406	0.5708	−0.0230	0.182*	
O1B	1.1025 (2)	0.65955 (17)	0.62049 (11)	0.0913 (6)	
O2B	0.6969 (2)	0.70067 (16)	0.44345 (12)	0.0897 (5)	
O3B	0.66014 (18)	0.91587 (15)	0.43543 (10)	0.0793 (5)	
C1B	1.0365 (2)	0.84866 (19)	0.67860 (13)	0.0600 (5)	
C2B	1.0984 (2)	0.9478 (2)	0.73535 (15)	0.0707 (6)	
H2B	1.1546	0.9356	0.7788	0.085*	
C3B	1.0797 (3)	1.0672 (2)	0.72990 (16)	0.0745 (6)	
C4B	0.9920 (2)	1.0823 (2)	0.66790 (14)	0.0670 (6)	
H4B	0.9785	1.1604	0.6635	0.080*	
C5B	0.9211 (2)	0.98404 (18)	0.61035 (13)	0.0563 (5)	
C6B	0.94478 (19)	0.86463 (18)	0.61375 (12)	0.0549 (5)	
C7B	0.8703 (2)	0.7674 (2)	0.55581 (13)	0.0615 (5)	
H7B	0.8854	0.6885	0.5565	0.074*	
C8B	0.7773 (2)	0.7878 (2)	0.49950 (14)	0.0646 (5)	
C9B	0.7553 (2)	0.9087 (2)	0.49535 (13)	0.0619 (5)	
C10B	0.8264 (2)	1.0031 (2)	0.54951 (13)	0.0602 (5)	
H10B	0.8126	1.0817	0.5466	0.072*	
C11B	1.1522 (4)	1.1752 (3)	0.7944 (2)	0.1160 (12)	
H11C	1.0838	1.2214	0.8207	0.139*	
H11D	1.1959	1.1441	0.8385	0.139*	
C12B	1.2469 (6)	1.2507 (4)	0.7619 (3)	0.175 (2)	
H12D	1.2870	1.3150	0.8062	0.262*	
H12E	1.2045	1.2843	0.7194	0.262*	
H12F	1.3167	1.2067	0.7371	0.262*	
C13B	1.0760 (2)	0.7286 (2)	0.68383 (15)	0.0654 (5)	
C14B	1.0863 (2)	0.69243 (19)	0.76813 (14)	0.0641 (5)	
C15B	1.1802 (3)	0.6170 (2)	0.78166 (19)	0.0832 (7)	
H15B	1.2376	0.5917	0.7387	0.100*	
C16B	1.1886 (4)	0.5797 (3)	0.8586 (2)	0.1031 (10)	
H16B	1.2533	0.5310	0.8676	0.124*	
C17B	1.1027 (4)	0.6135 (3)	0.9214 (2)	0.1047 (10)	
H17B	1.1088	0.5879	0.9730	0.126*	
C18B	1.0082 (3)	0.6850 (3)	0.90836 (17)	0.0912 (8)	
H18B	0.9483	0.7064	0.9507	0.109*	
C19B	1.0006 (3)	0.7259 (2)	0.83253 (15)	0.0728 (6)	
H19B	0.9372	0.7764	0.8249	0.087*	
C20B	0.6381 (3)	1.0335 (3)	0.42544 (18)	0.0891 (8)	
H20D	0.5691	1.0270	0.3819	0.134*	
H20E	0.7217	1.0775	0.4100	0.134*	
H20F	0.6088	1.0756	0.4777	0.134*	
C21B	0.7066 (4)	0.5784 (3)	0.4460 (2)	0.1110 (11)	
H21D	0.6452	0.5267	0.4038	0.166*	
H21E	0.6827	0.5614	0.5009	0.166*	
H21F	0.7985	0.5639	0.4350	0.166*	

### Atomic displacement parameters (Å^2^)

**Table:**

	*U*^11^	*U*^22^	*U*^33^	*U*^12^	*U*^13^	*U*^23^
O1A	0.1024 (13)	0.0857 (12)	0.0727 (11)	−0.0220 (10)	−0.0048 (10)	0.0054 (9)
O2A	0.1159 (14)	0.0642 (10)	0.0920 (12)	0.0127 (10)	0.0357 (11)	−0.0028 (9)
O3A	0.0804 (11)	0.0747 (10)	0.0719 (10)	0.0083 (8)	0.0221 (8)	0.0101 (8)
C1A	0.0552 (11)	0.0585 (11)	0.0594 (11)	0.0050 (9)	−0.0003 (9)	0.0126 (9)
C2A	0.0607 (12)	0.0644 (13)	0.0673 (13)	0.0084 (10)	0.0111 (10)	0.0161 (10)
C3A	0.0695 (13)	0.0597 (12)	0.0679 (13)	0.0129 (10)	0.0111 (11)	0.0092 (10)
C4A	0.0617 (12)	0.0508 (11)	0.0663 (12)	0.0064 (9)	0.0032 (10)	0.0099 (9)
C5A	0.0514 (10)	0.0535 (10)	0.0556 (11)	0.0082 (8)	−0.0021 (8)	0.0117 (8)
C6A	0.0544 (11)	0.0541 (11)	0.0543 (11)	0.0074 (8)	−0.0037 (8)	0.0100 (8)
C7A	0.0692 (13)	0.0531 (11)	0.0650 (12)	0.0055 (10)	0.0021 (10)	0.0093 (9)
C8A	0.0740 (14)	0.0572 (12)	0.0643 (13)	0.0139 (10)	0.0049 (11)	0.0046 (10)
C9A	0.0599 (12)	0.0650 (12)	0.0561 (11)	0.0098 (10)	0.0044 (9)	0.0127 (9)
C10A	0.0564 (11)	0.0546 (11)	0.0584 (11)	0.0069 (9)	−0.0001 (9)	0.0132 (9)
C11A	0.140 (3)	0.0658 (16)	0.113 (2)	0.0221 (17)	0.059 (2)	0.0075 (15)
C12A	0.400 (10)	0.086 (3)	0.286 (7)	−0.026 (4)	0.246 (8)	−0.049 (4)
C13A	0.0595 (12)	0.0598 (12)	0.0693 (13)	0.0007 (10)	0.0000 (10)	0.0087 (10)
C14A	0.0607 (12)	0.0509 (11)	0.0720 (13)	0.0039 (9)	0.0040 (10)	0.0116 (9)
C15A	0.0696 (14)	0.0587 (13)	0.0935 (17)	−0.0024 (11)	0.0076 (12)	0.0131 (12)
C16A	0.099 (2)	0.0681 (15)	0.112 (2)	−0.0038 (14)	0.0287 (18)	0.0302 (15)
C17A	0.132 (3)	0.0720 (16)	0.092 (2)	0.0124 (17)	0.0279 (19)	0.0343 (15)
C18A	0.122 (2)	0.0728 (16)	0.0755 (16)	0.0129 (16)	−0.0027 (15)	0.0212 (13)
C19A	0.0747 (15)	0.0638 (13)	0.0792 (15)	0.0031 (11)	−0.0011 (12)	0.0172 (11)
C20A	0.0834 (17)	0.0832 (17)	0.0845 (17)	0.0066 (13)	0.0219 (13)	0.0234 (13)
C21A	0.158 (3)	0.0627 (16)	0.132 (3)	0.0075 (18)	0.050 (2)	−0.0104 (17)
O1B	0.1117 (14)	0.0941 (13)	0.0770 (11)	0.0484 (11)	0.0196 (10)	0.0116 (9)
O2B	0.1035 (13)	0.0690 (11)	0.0878 (12)	0.0079 (9)	−0.0265 (10)	−0.0044 (9)
O3B	0.0847 (11)	0.0782 (11)	0.0740 (10)	0.0153 (9)	−0.0169 (9)	0.0097 (8)
C1B	0.0540 (11)	0.0652 (13)	0.0611 (12)	0.0101 (9)	0.0068 (9)	0.0107 (10)
C2B	0.0647 (13)	0.0735 (15)	0.0734 (14)	0.0067 (11)	−0.0065 (11)	0.0156 (11)
C3B	0.0752 (15)	0.0675 (14)	0.0756 (15)	−0.0011 (11)	−0.0105 (12)	0.0091 (11)
C4B	0.0717 (14)	0.0555 (12)	0.0726 (14)	0.0041 (10)	0.0030 (11)	0.0127 (10)
C5B	0.0526 (11)	0.0577 (11)	0.0577 (11)	0.0052 (9)	0.0095 (9)	0.0101 (9)
C6B	0.0504 (10)	0.0584 (11)	0.0562 (11)	0.0085 (9)	0.0110 (8)	0.0100 (9)
C7B	0.0642 (12)	0.0576 (12)	0.0625 (12)	0.0130 (10)	0.0066 (10)	0.0073 (9)
C8B	0.0681 (13)	0.0626 (13)	0.0592 (12)	0.0084 (10)	0.0025 (10)	0.0014 (10)
C9B	0.0613 (12)	0.0697 (13)	0.0559 (11)	0.0130 (10)	0.0042 (9)	0.0115 (10)
C10B	0.0610 (12)	0.0593 (12)	0.0625 (12)	0.0108 (9)	0.0088 (10)	0.0151 (10)
C11B	0.117 (3)	0.089 (2)	0.135 (3)	−0.0251 (19)	−0.046 (2)	0.0359 (19)
C12B	0.198 (5)	0.159 (4)	0.140 (4)	−0.060 (4)	−0.010 (3)	0.025 (3)
C13B	0.0579 (12)	0.0688 (13)	0.0704 (14)	0.0158 (10)	0.0055 (10)	0.0101 (11)
C14B	0.0596 (12)	0.0592 (12)	0.0714 (13)	0.0061 (10)	−0.0050 (10)	0.0088 (10)
C15B	0.0713 (15)	0.0776 (16)	0.104 (2)	0.0190 (13)	−0.0036 (14)	0.0193 (14)
C16B	0.099 (2)	0.094 (2)	0.124 (3)	0.0151 (17)	−0.029 (2)	0.039 (2)
C17B	0.127 (3)	0.095 (2)	0.090 (2)	−0.005 (2)	−0.030 (2)	0.0329 (17)
C18B	0.113 (2)	0.0872 (19)	0.0687 (16)	−0.0020 (17)	0.0014 (15)	0.0141 (14)
C19B	0.0759 (15)	0.0699 (14)	0.0714 (14)	0.0101 (12)	0.0004 (12)	0.0103 (11)
C20B	0.0974 (19)	0.0909 (19)	0.0831 (17)	0.0203 (15)	−0.0154 (15)	0.0227 (14)
C21B	0.134 (3)	0.0674 (17)	0.119 (2)	0.0113 (17)	−0.033 (2)	−0.0134 (16)

### Geometric parameters (Å, º)

**Table:**

O1A—C13A	1.222 (3)	O1B—C13B	1.225 (3)
O2A—C8A	1.365 (3)	O2B—C8B	1.366 (3)
O2A—C21A	1.418 (3)	O2B—C21B	1.412 (3)
O3A—C9A	1.363 (3)	O3B—C9B	1.362 (3)
O3A—C20A	1.419 (3)	O3B—C20B	1.419 (3)
C1A—C2A	1.371 (3)	C1B—C2B	1.378 (3)
C1A—C6A	1.426 (3)	C1B—C6B	1.429 (3)
C1A—C13A	1.498 (3)	C1B—C13B	1.487 (3)
C2A—C3A	1.402 (3)	C2B—C3B	1.411 (3)
C2A—H2A	0.9300	C2B—H2B	0.9300
C3A—C4A	1.374 (3)	C3B—C4B	1.365 (3)
C3A—C11A	1.507 (3)	C3B—C11B	1.541 (4)
C4A—C5A	1.412 (3)	C4B—C5B	1.409 (3)
C4A—H4A	0.9300	C4B—H4B	0.9300
C5A—C6A	1.417 (3)	C5B—C10B	1.415 (3)
C5A—C10A	1.417 (3)	C5B—C6B	1.418 (3)
C6A—C7A	1.419 (3)	C6B—C7B	1.420 (3)
C7A—C8A	1.360 (3)	C7B—C8B	1.357 (3)
C7A—H7A	0.9300	C7B—H7B	0.9300
C8A—C9A	1.425 (3)	C8B—C9B	1.432 (3)
C9A—C10A	1.361 (3)	C9B—C10B	1.357 (3)
C10A—H10A	0.9300	C10B—H10B	0.9300
C11A—C12A	1.290 (5)	C11B—C12B	1.349 (5)
C11A—H11A	0.9700	C11B—H11C	0.9700
C11A—H11B	0.9700	C11B—H11D	0.9700
C12A—H12A	0.9600	C12B—H12D	0.9600
C12A—H12B	0.9600	C12B—H12E	0.9600
C12A—H12C	0.9600	C12B—H12F	0.9600
C13A—C14A	1.493 (3)	C13B—C14B	1.490 (3)
C14A—C15A	1.391 (3)	C14B—C19B	1.380 (3)
C14A—C19A	1.392 (3)	C14B—C15B	1.392 (3)
C15A—C16A	1.378 (4)	C15B—C16B	1.381 (4)
C15A—H15A	0.9300	C15B—H15B	0.9300
C16A—C17A	1.372 (4)	C16B—C17B	1.366 (5)
C16A—H16A	0.9300	C16B—H16B	0.9300
C17A—C18A	1.382 (4)	C17B—C18B	1.362 (5)
C17A—H17A	0.9300	C17B—H17B	0.9300
C18A—C19A	1.375 (3)	C18B—C19B	1.384 (4)
C18A—H18A	0.9300	C18B—H18B	0.9300
C19A—H19A	0.9300	C19B—H19B	0.9300
C20A—H20A	0.9600	C20B—H20D	0.9600
C20A—H20B	0.9600	C20B—H20E	0.9600
C20A—H20C	0.9600	C20B—H20F	0.9600
C21A—H21A	0.9600	C21B—H21D	0.9600
C21A—H21B	0.9600	C21B—H21E	0.9600
C21A—H21C	0.9600	C21B—H21F	0.9600
			
C8A—O2A—C21A	118.0 (2)	C8B—O2B—C21B	117.7 (2)
C9A—O3A—C20A	117.46 (18)	C9B—O3B—C20B	117.09 (19)
C2A—C1A—C6A	119.87 (18)	C2B—C1B—C6B	119.9 (2)
C2A—C1A—C13A	118.13 (19)	C2B—C1B—C13B	117.9 (2)
C6A—C1A—C13A	121.81 (19)	C6B—C1B—C13B	122.12 (19)
C1A—C2A—C3A	122.6 (2)	C1B—C2B—C3B	122.3 (2)
C1A—C2A—H2A	118.7	C1B—C2B—H2B	118.8
C3A—C2A—H2A	118.7	C3B—C2B—H2B	118.8
C4A—C3A—C2A	117.7 (2)	C4B—C3B—C2B	117.7 (2)
C4A—C3A—C11A	123.1 (2)	C4B—C3B—C11B	121.9 (2)
C2A—C3A—C11A	119.2 (2)	C2B—C3B—C11B	120.4 (2)
C3A—C4A—C5A	122.08 (19)	C3B—C4B—C5B	122.6 (2)
C3A—C4A—H4A	119.0	C3B—C4B—H4B	118.7
C5A—C4A—H4A	119.0	C5B—C4B—H4B	118.7
C4A—C5A—C6A	119.43 (18)	C4B—C5B—C10B	120.8 (2)
C4A—C5A—C10A	120.66 (18)	C4B—C5B—C6B	119.6 (2)
C6A—C5A—C10A	119.92 (18)	C10B—C5B—C6B	119.65 (19)
C5A—C6A—C7A	118.08 (18)	C5B—C6B—C7B	118.24 (19)
C5A—C6A—C1A	118.12 (18)	C5B—C6B—C1B	117.91 (18)
C7A—C6A—C1A	123.79 (18)	C7B—C6B—C1B	123.74 (19)
C8A—C7A—C6A	121.1 (2)	C8B—C7B—C6B	121.0 (2)
C8A—C7A—H7A	119.5	C8B—C7B—H7B	119.5
C6A—C7A—H7A	119.5	C6B—C7B—H7B	119.5
C7A—C8A—O2A	125.5 (2)	C7B—C8B—O2B	125.6 (2)
C7A—C8A—C9A	120.6 (2)	C7B—C8B—C9B	120.6 (2)
O2A—C8A—C9A	113.9 (2)	O2B—C8B—C9B	113.8 (2)
C10A—C9A—O3A	125.74 (19)	C10B—C9B—O3B	126.2 (2)
C10A—C9A—C8A	119.72 (19)	C10B—C9B—C8B	119.4 (2)
O3A—C9A—C8A	114.54 (19)	O3B—C9B—C8B	114.34 (19)
C9A—C10A—C5A	120.62 (19)	C9B—C10B—C5B	121.1 (2)
C9A—C10A—H10A	119.7	C9B—C10B—H10B	119.5
C5A—C10A—H10A	119.7	C5B—C10B—H10B	119.5
C12A—C11A—C3A	124.0 (3)	C12B—C11B—C3B	114.9 (4)
C12A—C11A—H11A	106.3	C12B—C11B—H11C	108.5
C3A—C11A—H11A	106.3	C3B—C11B—H11C	108.5
C12A—C11A—H11B	106.3	C12B—C11B—H11D	108.5
C3A—C11A—H11B	106.3	C3B—C11B—H11D	108.5
H11A—C11A—H11B	106.4	H11C—C11B—H11D	107.5
C11A—C12A—H12A	109.5	C11B—C12B—H12D	109.5
C11A—C12A—H12B	109.5	C11B—C12B—H12E	109.5
H12A—C12A—H12B	109.5	H12D—C12B—H12E	109.5
C11A—C12A—H12C	109.5	C11B—C12B—H12F	109.5
H12A—C12A—H12C	109.5	H12D—C12B—H12F	109.5
H12B—C12A—H12C	109.5	H12E—C12B—H12F	109.5
O1A—C13A—C14A	119.39 (19)	O1B—C13B—C1B	121.4 (2)
O1A—C13A—C1A	121.2 (2)	O1B—C13B—C14B	119.3 (2)
C14A—C13A—C1A	119.40 (18)	C1B—C13B—C14B	119.27 (19)
C15A—C14A—C19A	119.3 (2)	C19B—C14B—C15B	118.3 (2)
C15A—C14A—C13A	118.4 (2)	C19B—C14B—C13B	122.2 (2)
C19A—C14A—C13A	122.33 (19)	C15B—C14B—C13B	119.4 (2)
C16A—C15A—C14A	119.9 (3)	C16B—C15B—C14B	120.3 (3)
C16A—C15A—H15A	120.0	C16B—C15B—H15B	119.9
C14A—C15A—H15A	120.0	C14B—C15B—H15B	119.9
C17A—C16A—C15A	120.4 (3)	C17B—C16B—C15B	120.5 (3)
C17A—C16A—H16A	119.8	C17B—C16B—H16B	119.8
C15A—C16A—H16A	119.8	C15B—C16B—H16B	119.8
C16A—C17A—C18A	120.1 (3)	C18B—C17B—C16B	119.8 (3)
C16A—C17A—H17A	120.0	C18B—C17B—H17B	120.1
C18A—C17A—H17A	120.0	C16B—C17B—H17B	120.1
C19A—C18A—C17A	120.1 (3)	C17B—C18B—C19B	120.5 (3)
C19A—C18A—H18A	119.9	C17B—C18B—H18B	119.8
C17A—C18A—H18A	119.9	C19B—C18B—H18B	119.8
C18A—C19A—C14A	120.1 (2)	C14B—C19B—C18B	120.6 (3)
C18A—C19A—H19A	120.0	C14B—C19B—H19B	119.7
C14A—C19A—H19A	120.0	C18B—C19B—H19B	119.7
O3A—C20A—H20A	109.5	O3B—C20B—H20D	109.5
O3A—C20A—H20B	109.5	O3B—C20B—H20E	109.5
H20A—C20A—H20B	109.5	H20D—C20B—H20E	109.5
O3A—C20A—H20C	109.5	O3B—C20B—H20F	109.5
H20A—C20A—H20C	109.5	H20D—C20B—H20F	109.5
H20B—C20A—H20C	109.5	H20E—C20B—H20F	109.5
O2A—C21A—H21A	109.5	O2B—C21B—H21D	109.5
O2A—C21A—H21B	109.5	O2B—C21B—H21E	109.5
H21A—C21A—H21B	109.5	H21D—C21B—H21E	109.5
O2A—C21A—H21C	109.5	O2B—C21B—H21F	109.5
H21A—C21A—H21C	109.5	H21D—C21B—H21F	109.5
H21B—C21A—H21C	109.5	H21E—C21B—H21F	109.5

## References

[bb1] Alvarez, C., Alvarez, R., Corchete, P., Perez-Melero, C., Pelaez, R. & Medarde, M. (2007). *Bioorg. Med. Chem. Lett.* **17**, 3417–3420. 10.1016/j.bmcl.2007.03.082 17434303

[bb2] Bruker (2004). *APEX2* and *SAINT* Bruker AXS Inc., Madison, Wisconsin, USA.

[bb3] Cremer, D. & Pople, J. A. (1975). *J. Am. Chem. Soc.* **97**, 1354–1358.

[bb4] Dekoning, C. B., Rousseau, A. L. & Vanotterlo, W. A. (2003). *Tetrahedron*, **59**, 7–36.

[bb5] Farrugia, L. J. (1997). *J. Appl. Cryst.* **30**, 565.

[bb6] Nardelli, M. (1995). *J. Appl. Cryst.* **28**, 659.

[bb7] Parkin, S., Moezzi, B. & Hope, H. (1995). *J. Appl. Cryst.* **28**, 53–56.

[bb8] Sheldrick, G. M. (2008). *Acta Cryst.* A**64**, 112–122. 10.1107/S0108767307043930 18156677

[bb9] Spek, A. L. (2009). *Acta Cryst.* D**65**, 148–155. 10.1107/S090744490804362X PMC2631630 19171970

[bb10] Thenmozhi, S., SubbiahPandi, A., Ranjith, S., Clement, J. A. & Mohana­Krishnan, A. K. (2008). *Acta Cryst.* E**64**, o2432. 10.1107/S1600536808038701 PMC2959828 21581400

[bb11] Watanabe, S., Nakaema, K., Nishijima, T., Okamoto, A. & Yonezawa, N. (2010). *Acta Cryst.* E**66**, o615. 10.1107/S1600536810005398 PMC2983576 21580373

